# Combination Adjuvants Enhance Recombinant H5 Hemagglutinin Vaccine Protection Against High-Dose Viral Challenge in Chickens

**DOI:** 10.3390/vaccines12121448

**Published:** 2024-12-23

**Authors:** Yanjuan He, Jiaxin Wang, Lanyan Chi, Yajing Dong, Huixin Chen, Xiaocui Meng, Ming Liao, Yongwen Luo, Huiying Fan

**Affiliations:** 1 College of Veterinary Medicine, South China Agricultural University, Guangzhou 510642, China; 17818521991@163.com (Y.H.); wangjiaxinlalala@163.com (J.W.); 13713868212@163.com (L.C.); dyj@stu.scau.edu.cn (Y.D.); chenhuixin2023@stu.scau.edu.cn (H.C.); 20233073107@stu.scau.edu.cn (X.M.); mliao@scau.edu.cn (M.L.); 2 Key Laboratory of Veterinary Vaccine Innovation of the Ministry of Agriculture and Rural Affairs, Guangzhou 510642, China; 3 Key Laboratory of Zoonosis Prevention and Control of Guangdong Province, Guangzhou 510642, China; 4 National and Regional Joint Engineering Laboratory for Medicament of Zoonosis Prevention and Control, Guangzhou 510642, China

**Keywords:** avian influenza, H5 subtype, adjuvanted vaccine, Quil-A, TLR agonist

## Abstract

Background: Recombinant avian influenza subunit vaccines often require adjuvants to enhance immune responses. This study aims to evaluate the immune-enhancing potential of seven combination adjuvants in specific pathogen-free (SPF) chickens. Methods: SPF chickens were vaccinated with combinations of ISA78VG and adjuvants, including Quil-A, CpG, and monophosphoryl lipid A (MPLA). Their immune responses were assessed using a vaccination and viral challenge protection model. Results: The combinations of ISA78VG with Quil-A, CpG&MPLA or CpG&Quil-A significantly enhanced antibody responses and provided cross-protection against the H5N8-20135 strain. The ISA78VG&MPLA and ISA78VG&CpG&MPLA combinations induced the stronger IFN-γ production, with CpG further amplifying the immune response. The ISA78VG&Quil-A formulation, in particular, stimulated rapid antibody responses, achieving a 100% seroconversion by day 14 and high titers of hemagglutination inhibition (HI) antibodies against both the recombinant HA antigen and the H5N6-20053 virus. Conclusions: The ISA78VG&Quil-A combination is an ideal adjuvant for enhancing the immunogenicity of avian influenza rHA subunit vaccines, offering a promising strategy for H5 subtype vaccine development.

## 1. Introduction

Avian influenza (AI) is a highly contagious respiratory disease caused by the avian influenza virus (AIV). Since 2013, the H5N6 subtype of AIV has been circulating in Chinese poultry populations within clade 2.3.4.4 [1]. In 2014, highly pathogenic H5N6 outbreaks were reported on multiple continents [2]. Wild birds can harbor AIV, which not only triggers AI outbreaks but also poses a risk of human infection. As of 12 March 2024, China has reported two human cases of AI (H5N6) infection [3], underscoring the potential for H5N6 AIV to pose an epidemic threat.

Vaccination remains a key strategy for controlling the spread of AI. The most widely used AI vaccine is the whole-virus inactivated vaccine [4,5], but concerns over allergic reactions and pathogen contamination have driven research into alternative approaches [6,7,8]. Subunit vaccines, which focus on specific microbial proteins, have been extensively studied as a potential solution [9]. The influenza hemagglutinin HA protein is a major viral glycoprotein and has been the primary target antigen for vaccine development [10,11]. However, the immunogenicity of the H5 subtype HA antigen is relatively weak, underscoring the need for adjuvants to enhance vaccine efficacy [12,13].

Adjuvants enhance the immune response to antigens by facilitating their delivery to immune cells, thereby boosting the body’s natural defenses [14]. As novel adjuvanted vaccines have emerged, single-function adjuvants are no longer sufficient to meet the growing demands for immunogenicity. Instead, synergistic combinations of different adjuvants are being used to amplify immune responses. Quil-A, a mixture of saponins derived from the Quillaja plant, is primarily used in veterinary vaccines [15,16]. It has been included as an adjuvant in several clinical trials, including the Quil-A-containing Nuvaxovid SARS-CoV-2 vaccine, which has been approved in several countries, including the United States and Canada [17,18]. Toll-like receptors (TLRs) are essential pathogen sensors that recognize and bind to microbe-associated molecular patterns presented by both pathogenic and non-pathogenic microorganisms. Additionally, they play a crucial role in sensing damage-associated molecular patterns (DAMPs). Consequently, TLRs have attracted significant interest as potential targets for vaccine adjuvants [19,20,21]. CpG oligodeoxynucleotides (CpG ODN), TLR9 agonists, induce immunomodulatory effects and have been approved as adjuvants in the hepatitis B vaccine in the United States [22]. Although TLR9 is absent in the chicken genome, studies have demonstrated that chickens can mount an immune response to CpG ODN [23]. The TLR4 agonist monophosphoryl lipid A (MPLA), which promotes cytokine production and T-cell activation, has been approved for use in adjuvant systems for vaccines against several diseases, such as AS01, AS02, and AS04 [24,25,26]. Imiquimod, a TLR7/8 agonist that recognizes single-stranded RNA, is another adjuvant with potential [27]. While adjuvants like CpG ODN, MPLA, and Imiquimod are widely used in human vaccines, their role in veterinary vaccines remains largely unexplored.

Montanide ISA78VG, a mineral oil adjuvant under development, has shown promise as an effective additive for poultry vaccine formulations. Building upon this foundation, we designed and prepared novel compound adjuvants combining single or dual components (CpG, Quil-A, MPLA, Imiquimod, CpG&MPLA, and CpG&Quil-A). These compounds were evaluated for their ability to enhance antigen immunogenicity and induce immunity in poultry through challenge protection testing. Our findings support the potential of Quil-A-based formulations in advancing poultry vaccine adjuvants.

## 2. Materials and Methods

### 2.1. Cells and Viruses

MDCK cells, obtained from our laboratory, were cultured at 37 °C with 5% CO_2_ using DMEM supplemented with 10% heat-inactivated fetal bovine serum (Invitrogen, Carlsbad, CA, USA). Spodoptera frugiperda 9 (sf9) insect cells, also obtained from our laboratory, were cultured in an Sf-SFM serum-free medium (Womei Bio, Suzhou, China) at 27 °C. Two highly pathogenic avian H5 AIV strains (H5N6-20053 and H5N8-20135) originated from clade 2.3.4.4h (A/Duck/Fujian/20053/2020) and 2.3.4.4b (A/Duck/Sicuan/20135/2020), and they were used in the detection of hemagglutination Inhibition (HI) and virus neutralization (VN) antibodies. The viruses are maintained in a laboratory. H5N6-20053 was used as a source of the HA gene and for viral challenges.

### 2.2. Recombinant Antigen and Adjuvants

The rHA protein of H5N6-20053 was expressed in sf9 cells using the baculovirus expression system [28,29], and the hemagglutination activity is illustrated in Figure S1. The sequence of CpG-1018 was 5′-TGACTGTGAACGTTCGAGATGA-3′, which was synthesized by Sangon Bioengineering (Shanghai, China). The reagents Quil-A, MPLA and Imiquimod were obtained from Invitrogen. ISA78VG was provided by Seppic (Paris, France).

### 2.3. Immuization and Viral Challenge Protection Trials

Three-week-old SPF chickens were purchased from the Laboratory Animal Center (Guangdong, China). They were maintained according to the guidelines for the care and use of laboratory animals by the South China Agricultural University. The immunogenicity and vaccination efficacy of the combination adjuvants against H5N6-20053 rHA were examined in two independent experiments (Exp 1 and Exp 2). In Exp 1, a total of 78 SPF chickens were used to conduct a challenge protection study with 10^6^ EID_50_ in different combination adjuvant immunization groups. Each combination adjuvant immunization group received a single subcutaneous neck immunization with 50 μg H5N6-20053 rHA mixed with various adjuvants (*n* = 10 per group), administered at a volume of 300 μL. The ISA 78 immunization group and the PBS immunization group served as the vaccine control and negative control groups. Blood samples of 1mL were collected from all test chickens via the jugular vein at 2 and 3 weeks post-vaccination, and the serum was separated to detect the antibody levels. Three weeks post-immunization, the chickens were intranasally inoculated with 10^6^ EID_50_ (0.2 mL) of highly pathogenic AIV H5N6-20053. Oropharyngeal and cloacal swabs were collected on days 3, 5 and 7. The details of the antigen and adjuvant preparation scheme can be found in Table 1. In Exp 2, a total of 15 SPF chickens were used to further assess the efficacy of immunity, comparing the efficacy of ISA78VG&Quil-A and ISA78VG&MPLA in providing protection against a high dose (10^8^ EID_50_/0.2mL) challenge. The antigens and adjuvants used in the ISA78VG&Quil-A and ISA78VG&MPLA groups were consistent with those utilized in Exp 1. The PBS immunization group served as the negative control. In Exp 2, chickens (*n* = 5) were given a single subcutaneous injection in the neck for immunization, and the serum was collected and separated at 8, 10, 12, 14, 16 and 21 days post-vaccination, respectively. Three weeks post-vaccination, the chickens were intranasally inoculated with 10^8^ EID_50_ (0.2 mL) of the highly pathogenic AIV (H5N6-20053), and oropharyngeal and cloacal swabs were collected on day 5. The chickens were monitored for clinical signs and mortality for 2 weeks after the challenge. All surviving chickens were humanely killed at the end of the monitoring experiment.

### 2.4. Hemagglutination Inhibition Assay

The hemagglutination inhibition (HI) assay may be utilized to detect or quantify antibodies to the influenza A viruses and can be used to characterize differences in the antigenic reactivity between influenza isolates [30]. The serum samples were serially diluted two-fold with PBS in a 96-well V-shaped plate and incubated with 4 HA units (HAUs) of AIV H5N6-20053, AIV H5N8-20135, and H5N6-20053 rHA. The serum–virus mixture was incubated at room temperature for 40 min, and then incubated with 25 μL of 1% chicken red blood cells at room temperature for 30 min. The antibody titer is expressed as the reciprocal of the highest serum dilution showing the complete inhibition of hemagglutination.

### 2.5. Virus Neutralization Assay

The serums collected at 2 and 3 weeks post-immunization were used to measure virus neutralization antibodies. MDCK (2 × 10^5^ cells/mL) were seeded in a 96-well plate and incubated at 37 °C with 5% CO_2_ for 12 h. The sera were diluted in a solution containing 2 mg/mL BSA (Dingguo, Guangzhou, China) and 0.5 μg/mL TPCK-trypsin (Dingguo), with 2-week serums diluted 10-fold and 3-week serums diluted 40-fold. The diluted serums were serially diluted two-fold. Equal volumes of the diluted serums and 100 TCID_50_ of H5N6-20053 or H5N8-20135 virus were mixed and incubated for 72 h. Virus presence was detected using hemagglutination assays. The Virus Neutralization antibody (VNb) titer was defined as the reciprocal of the highest serum dilution at which 50% of the wells were protected from the AIV challenge.

### 2.6. Isolation of Chicken PBMCs and Detection of Antigen Stimulated Cytokines

Peripheral blood mononuclear cells (PBMCs) were isolated from peripheral blood using the Ficoll-Hypaque density sedimentation method (Tbd Science, Tianjin, China). PBMCs (1 × 10^6^ cells/mL) were inoculated into a 6-well plate. The cells were stimulated with 50 μg H5N6-20053 rHA protein and incubated at 37 °C for 6 h, then harvested for RNA extraction.

Cytokine mRNA levels were quantified using the ChamQ Universal SYBR qPCR Master Mix (Vazyme, Nanjing, China). The total RNA from stimulated cells was extracted with a Total RNA Rapid Extraction Kit (Feijie, Shanghai, China), and reverse transcription was performed using HiScript^®^ III RT SuperMix for qPCR (Vazyme, Nanjing, China). The qPCR was carried out in a 20 µL reaction mixture, which included 10 µL of 2× ChamQ Universal SYBR qPCR Master Mix, 0.4 µL of 10 µM forward and reverse primers, and 1 µL of cDNA. The amplification conditions were as follows: initial denaturation at 95 °C for 30 s, followed by 40 cycles of denaturation at 95 °C for 10 s, annealing at 60 °C for 30 s, and a post-melting curve analysis using the default parameters. The relative gene expression was calculated using the 2^−ΔΔCt^ method with β-actin as the internal control. The primer sequences for target genes are listed in Table 2.

### 2.7. Statistical Analysis

Experimental data are presented as mean ± standard deviation (SD). A statistical analysis was performed using SPSS version 27 software. A log-rank (Mantel–Cox) test was used to analyze the survival state after the challenge. Student’s *t*-tests were applied for comparing two independent datasets. GraphPad Prism version 9 software was used to plot the results. The statistical significance is denoted as follows: * *p* < 0.05, ** *p* < 0.01.

## 3. Results

### 3.1. Enhanced Antibody Response to H5N6-20053 rHA Vaccine with Combination Adjuvants

**Figure 1:**
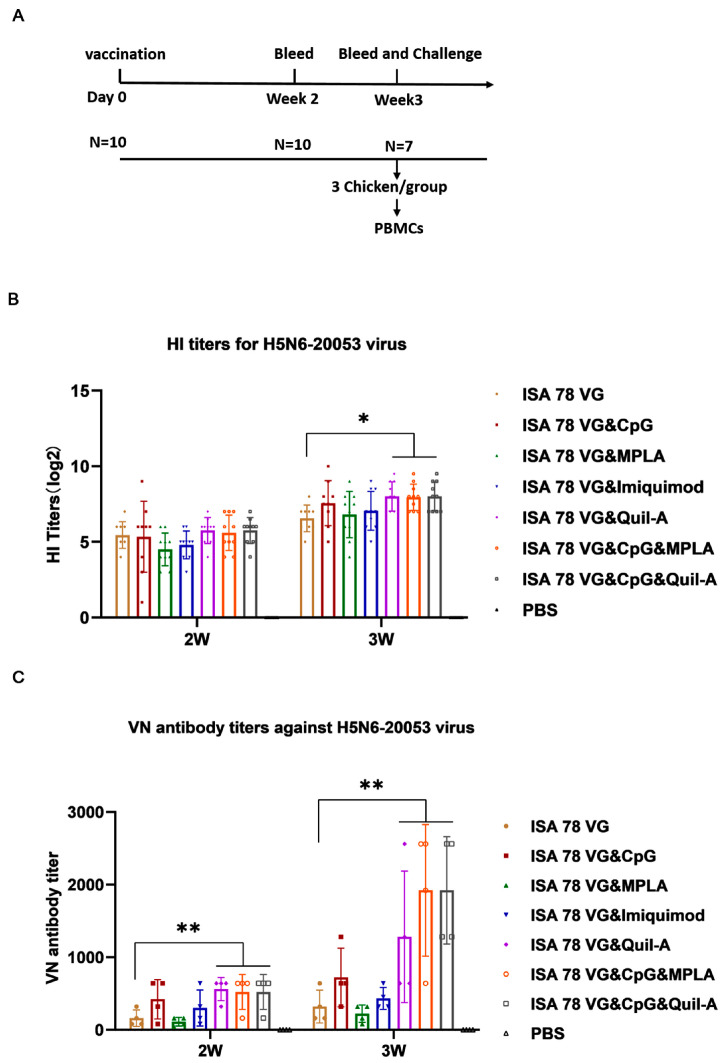
Immunization procedures and antibody levels against H5N6-20053 virus. (**A**) Animal experimental design for immunization and challenge. Serum samples from each group of vaccinated chickens were collected at 14 and 21 days post-vaccination. Viral challenge protection test against HPAI H5N6-20053 virus conducted 3 weeks post-immunization. (**B**) HI titers for H5N6-20053 virus. (**C**) Neutralizing antibodies against 100 TCID_50_ H5N6-20053 virus were measured using neutralization assays in each group (*n* = 4). Statistical significance of differences is illustrated as follows: * *p* < 0.05, ** *p* < 0.01.

**Figure 2:**
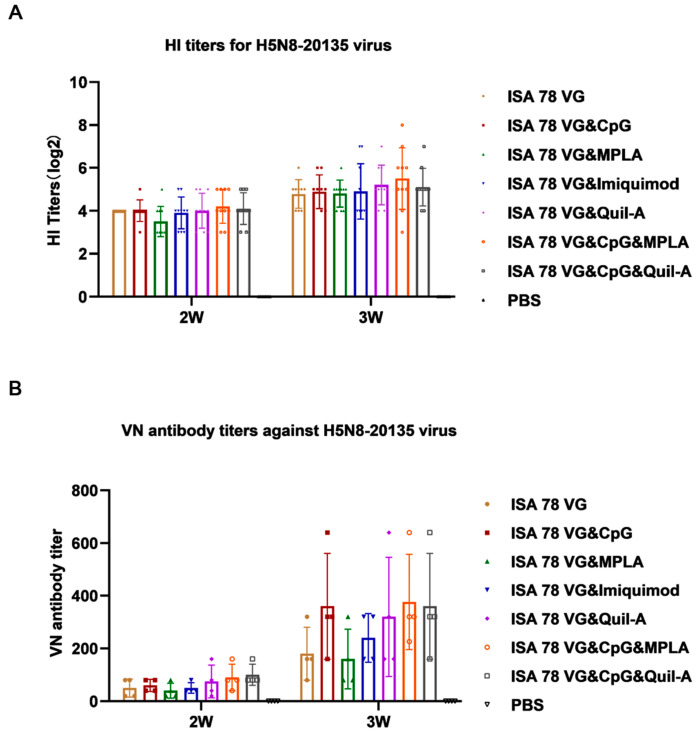
Antibody levels against H5N8-20135 virus. (**A**) HI titers for H5N8-20135 virus. (**B**) Neutralizing antibodies for 100 TCID_50_ H5N8-20135 virus.

To assess the effect of different combination adjuvants on the immunogenicity of H5N6-20053 rHA, SPF chickens were immunized with a single dose of 50 μg H5N6-20053 rHA protein combined with various adjuvant formulations (Table 1). The serum samples collected at 2 and 3 weeks post-vaccination were analyzed for HI and VN antibodies for AIV H5N6-20053 (Figure 1) and their cross-reactivity for AIV H5N8-20135 (Figure 2). In chickens infected with the H5 subtype AIV, common clinical signs included lethargy, respiratory distress (such as nasal discharge, coughing, and sneezing), swelling of the head and neck, and in some cases, diarrhea [31]. In our study, no adverse clinical signs were observed in the chickens following vaccination.

By week 3, all vaccinated chickens had developed high antibody levels against the H5N6-20053 virus. The ISA78VG&Quil-A, ISA78VG&CpG&MPLA, and ISA78VG&CpG&Quil-A groups had significantly higher HI titers (8 log2) than the ISA78VG group (6.6 log2) (Figure 1B). No significant differences were observed between the ISA78VG, ISA78VG&CpG, ISA78VG&MPLA, and ISA78VG&Imiquimod groups. Overall, the antibody responses from the ISA78VG&Quil-A, ISA78VG&CpG&MPLA, and ISA78VG&CpG&Quil-A formulations were higher than those from the commercial vaccine. Importantly, all vaccinated chickens also had HI titers greater than 4 log2 against the H5N8-20135 virus by week 3, indicating a broad cross-protection (Figure 2A).

VN antibody titers against the H5N6-20053 virus were significantly higher in all vaccinated groups, compared to the PBS control group at both weeks 2 and 3 (Figure 1C). Specifically, the ISA78VG&Quil-A group exhibited VN antibody titers of 1:560 and 1:1280, the ISA78VG&CpG&MPLA group showed titers of 1:520 and 1:1920, and the ISA78VG&CpG&Quil-A group had titers of 1:520 and 1:1920. Titers in these combination adjuvant groups were significantly higher than those in the ISA78VG group at both weeks 2 (1:160) and 3 (1:320) (*p* < 0.01). No significant differences were observed between the ISA78VG&CpG and ISA78VG&MPLA groups. As shown in Figure 2B, all vaccine groups produced VN antibodies against the H5N8-20135 virus, with titers increasing over time (week 3 > week 2). No significant differences were found in the VNb titers between adjuvant groups at either time point.

These results demonstrate that the vaccine induced strong antibody responses against both the H5N6-20053 and H5N8-20135 strains in all groups at 2 and 3 weeks post-vaccination, enhancing the immunogenicity of H5N6-20053 rHA in SPF chickens. The ISA78VG&Quil-A, ISA78VG&CpG&MPLA, and ISA78VG&CpG&Quil-A groups reached high levels of antibody titers in week 3.

### 3.2. Combination Adjuvants Enhanced Cytokine Production of H5N6-20053 rHA Vaccine

**Figure 3:**
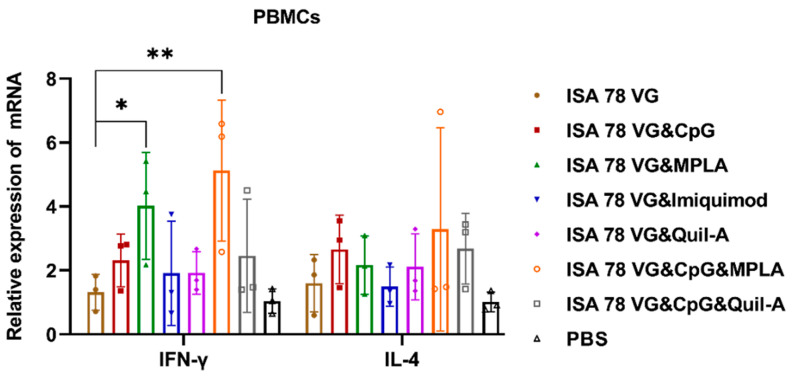
mRNA expression levels of cytokines in PBMCs. The expression levels of IFN-γ and IL-4 following antigen stimulation were quantified using qRT-PCR. Statistical comparisons between groups were conducted using the *t*-test, with the data presented as mean ± SD. Significance levels are denoted as follows: * *p*  <  0.05, ** *p*  <  0.01.

To evaluate the impact of various adjuvants on cytokine production, we measured the expression of IFN-γ and IL-4—using qRT-PCR (Figure 3). The IFN-γ mRNA levels were significantly higher in the ISA78VG&MPLA group compared to the ISA78VG single-adjuvant group, indicating the enhanced PBMC activation and IFN-γ secretion. The combination of CpG with ISA78VG&MPLA further boosted IFN-γ expression. Importantly, while IL-4 expression was detectable, IFN-γ mRNA levels in both the ISA78VG&MPLA and ISA78VG&CpG&MPLA groups consistently exceeded those of IL-4.

### 3.3. Efficacy of Combination Adjuvants in Inducing Protection Against H5N6-20053 Virus Challenge

**Figure 4:**
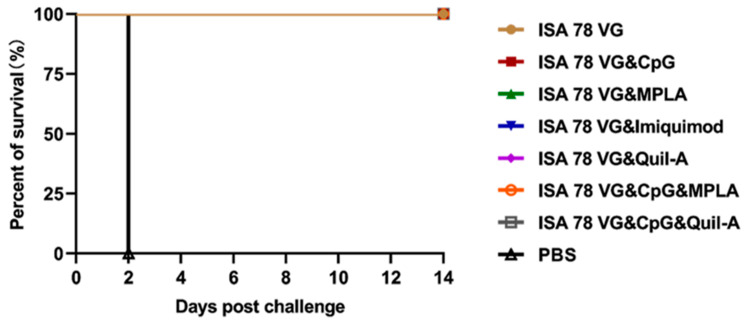
Survival rate of chickens after H5N6-20053 virus challenge: PBS group (*n* = 5) and vaccine group (*n* = 7).

To evaluate the protective efficacy of our vaccine candidate, the chickens were challenged with a lethal dose (10^6^ EID_50_) of H5N6-20053 virus at 3 weeks post-vaccination (Figure 4). All chickens in the adjuvant vaccine groups survived the 14-day challenge, achieving 100% survival. While mild behavioral and fecal changes were observed in some ISA78VG&Imiquimod group chickens, no significant clinical symptoms were noted in the other groups. In contrast, all chickens in the PBS control group succumbed to the infection within 2 days, exhibiting typical signs of illness, including lethargy, bradykinesia, and neurological disorders.

### 3.4. Combination Adjuvant Vaccines Reduce Viral Shedding and Replication in Chickens

Oropharyngeal and cloacal swabs of all chickens were collected on days 3, 5 and 7 after the challenge and were inoculated into 9-day-old chick embryos for virus isolation to evaluate the immunoprotective efficacy of different adjuvant vaccines. The results of the virus isolation in swabs are shown in Table 3.

Following the H5N6-20053 challenge, two chickens in the ISA78VG group shed viruses from oropharyngeal swabs on day 3. In the ISA78VG&Imiquimod group, three chickens shed viruses on days 3, 5, and 7, respectively. Notably, no viral shedding was detected in any of the other vaccine groups. These results indicate that all of the vaccine groups, except ISA78VG and ISA78VG&Imiquimod, provided a complete protection against the H5N6-20053 virus.

### 3.5. Rapid Antibody Response to ISA78VG&Quil-A Adjuvanted Vaccine in Immunized Chickens

Based on the above experimental results, we observed that the ISA78VG&Quil-A group exhibited higher serum antibody levels but lower cellular immunity, whereas the ISA78VG&MPLA group induced a TH1-biased cellular immune response with lower serum antibody levels. To further investigate, serums were collected on days 8, 10, 12, 14, and 16 post-vaccination, and the HI antibody positive conversion rate against H5N6-20053 was measured (Table 4).

The results showed that the HI antibodies in the ISA78VG&Quil-A group induced detectable antibody responses as early as day 10, achieving 100% seroconversion by day 14, while the ISA78VG&MPLA group did not reach full seroconversion by day 16. These findings suggest that the ISA78VG&Quil-A adjuvant induces an earlier antibody response compared to ISA78VG&MPLA.

### 3.6. Combination Adjuvant Vaccines Protect Against Higher Doses of H5N6-20053 Virus

**Figure 5:**
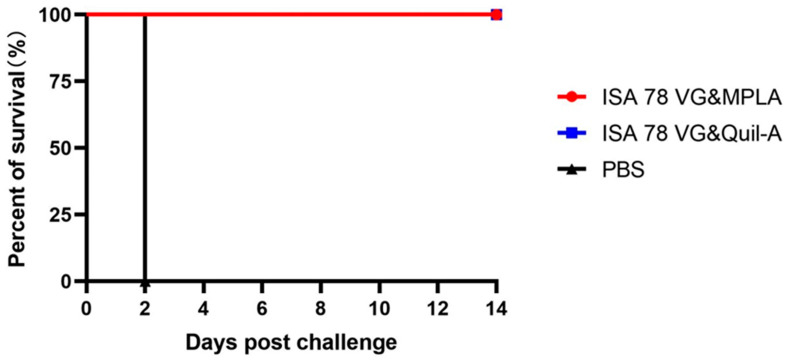
Survival rate of chickens tested after challenge. After the lethal H5N6-20053 virus (1 × 10^8^ EID_50_) challenge, the survival rates were compared between the PBS group (*n* = 5) and the vaccine group (*n* = 5).

Based on the above experimental results, we found that both ISA78VG&Quil-A and ISA78VG&MPLA combinations with H5N6-20053 rHA provided a complete protection against the lethal challenge. As shown in Table 5, the ISA78VG&Quil-A and ISA78VG&MPLA groups elicited HI titers of 9.8 log2 and 7.8 log2, respectively, following the vaccination with the recombinant H5 (rHA) antigen at 21 days post-vaccination. The corresponding HI titers against the challenge virus strain were 8 log2 and 6.8 log2, respectively. In contrast, the PBS control group exhibited HI titers of less than 4 log2 for the rHA antigen and 0 log2 for the challenge virus. Immunized chickens were challenged with a high dose (1 × 10^8^ EID_50_) of the virus and monitored for survival over 14 days (Figure 5). All PBS control chickens succumbed to the infection within 2 days, showing typical clinical symptoms, while all vaccinated chickens survived the challenge.

Oropharyngeal and cloacal swabs were collected on day 5, post-challenge, to assess virus shedding. The virus isolation results (Table 5) showed no viral shedding in any of the vaccine groups. These findings confirm that the combination adjuvant vaccines provide effective protection against a high-dose (1 × 10^8^ EID_50_) of the H5N6-20053 virus.

## 4. Discussion

The use of multiple adjuvants in a vaccine can enhance immune responses by activating both the humoral and cell-mediated immunity. As no single adjuvant can perform all these functions, we have evaluated various adjuvant combinations to improve vaccine efficacy [32,33]. In the present study, we evaluated various adjuvant combinations to enhance the immunogenicity of an H5N6-20053 rHA subunit vaccine for poultry. Specifically, we tested the combination of ISA78VG, a squalene-based oil emulsion, with TLR agonists (CpG ODNs and MPLA) and Quil-A, aiming to identify cost-effective and potent adjuvants that could improve vaccine performance.

Adjuvants can sometimes cause adverse reactions, including allergy, depression, loss of appetite, and local inflammation [34]. Serum HI antibody titers are closely related to AI prevention and serve as an important indicator of influenza vaccine antibody detection [35]. In this study, we prepared an H5N6-20053 rHA subunit vaccine with seven combination adjuvants and administered it subcutaneously to SPF chickens. A macroscopic observation showed no adverse effects, confirming the safety of the vaccine. Our results showed that combining ISA78VG with Quil-A, CpG&MPLA, or CpG&Quil-A significantly enhanced antigen immunogenicity and induced higher and more durable antibody responses. The HI antibody titers in the ISA78VG&Quil-A, ISA78VG&CpG&MPLA, and ISA78VG&CpG&Quil-A groups were significantly higher than in the ISA78VG group (*p* < 0.05), indicating that these adjuvant combinations enhanced the humoral immune response.

Interestingly, MPLA and Imiquimod have shown good immune effects in other vaccine trials and are widely used in vaccine development [36,37,38], but their effect is not significant in our data. In particular, the ISA78VG&Imiquimod group showed only a 42.9% protection rate, whereas other adjuvant combinations provided complete protection. These results suggest that Imiquimod negatively affected the immunogenicity of the ISA78VG vaccine, making the ISA78VG&Imiquimod combination less effective for H5N6-20053 rHA subunit vaccination. Similarly, the ISA78VG&MPLA group showed no significant improvement in antibody levels, compared to the use of ISA78VG alone. In some cases, the antibody levels were even lower in the ISA78VG&MPLA group, possibly due to the hydrophobic nature of MPLA, which may reduce the immune response when combined with the ISA78VG oil emulsion [39].

MPLA, as a TLR4 agonist, modulates immune responses by inducing the IFN-β (TRIF) pathway through adaptor molecules contained in the Toll/IL-1R domain [40]. In human studies, MPLA has been shown to promote protective Th1 responses while suppressing Th2 responses, primarily through the regulation of IL-10 and IL-12 [41]. CpG ODNs, which act as TLR9 agonists, stimulate B cells and plasmacytoid dendritic cells (pDCs), triggering the production of type I interferons and enhancing the overall immune response. The synergistic effect of CpG ODNs and MPLA can significantly increase IFN-γ levels, thereby promoting a robust Th1-type immune response [40]. In our study, this synergistic effect was observed, as the combination of CpG ODNs and MPLA elevated IFN-γ expression, as evidenced by our results. Our analysis of the cytokine profiles, specifically IL-4 and IFN-γ, showed that all adjuvant combinations induced the secretion of both IL-4 and IFN-γ. However, while IL-4 levels did not significantly differ between groups, IFN-γ expression was significantly higher in the ISA78VG&MPLA and ISA78VG&CpG&MPLA groups. These findings emphasize that the combination of adjuvants has the potential to induce IFN-γ production, which is crucial for enhancing cell-mediated immunity and providing long-lasting protection.

Although our study focused on evaluating the adjuvants’ effects on humoral immunity, we acknowledge the importance of cell-mediated immunity in providing long-term protection. In this study, due to the absence of specific cell population analyses, such as the isolation of CD4^+^ and CD8^+^ T cells, the role of cell-mediated immunity in our study remains unclear. Future studies should focus on isolating these cell populations to better understand their contribution to the observed immune responses and to provide a more comprehensive view of the immune mechanisms induced by these adjuvant combinations. Moreover, previous studies have shown that certain adjuvants, including mineral oil-based adjuvants [42], CpG, and MPLA [43], have the potential to prolong immune responses. Additionally, in earlier studies, cytokines were measured on day 19 post-immunization [29], which could explain the observed immune activation at the three-week time point in our study. This time point is essential for long-lasting protection. However, we recognize that the absence of a detailed cytokine profile at additional time points post-immunization represents a limitation of our study design. In future experiments, we plan to incorporate a more comprehensive cytokine analysis at various time points to further investigate the dynamics of the immune response and enhance the robustness of our conclusions.

The immunological protection from influenza vaccines relies on both humoral and cellular immune responses [44]. In our adjuvant screening, we found that Quil-A primarily enhances humoral immunity, while MPLA favors a Th1 response. High-potency vaccines that provide early protection are crucial for controlling disease spread and mutant strains [45]. Our research demonstrates that the ISA78VG&Quil-A group showed enhanced humoral immunity on day 10 post-vaccination, with HI antibody titers reaching 8 log2 by week 3. This suggests that Quil A mainly accelerates and intensifies the humoral immune response induced by the ISA78VG adjuvant vaccine in SPF chickens. These findings indicate that ISA78VG&Quil-A enhances early antibody responses to oil-adjuvanted H5N6-20053 rHA vaccines in SPF chickens.

This study aimed to identify an optimal composite adjuvant for the H5N6-20053 rHA protein, providing valuable insights for the development of AI vaccines. The novel adjuvants ISA78VG&Quil-A, ISA78VG&CpG&MPLA, and ISA78VG&CpG&Quil-A demonstrated antigen-presenting and immune-enhancing properties. The impact of combining different adjuvants on manufacturing costs needs to be thoroughly evaluated before they can be used to produce commercial animal vaccines [46]. Liu et al. have successfully synthesized QS-21 in yeast, significantly reducing production costs [47]. Considering both efficacy and cost, we propose that ISA78VG&Quil-A is a highly suitable, cost-effective adjuvant for the H5N6-20053 rHA subunit vaccine. In conclusion, our study identifies several adjuvant combinations that enhance the immunogenicity of an H5N6-20053 rHA subunit vaccine in poultry, with ISA78VG&Quil-A being the most promising in terms of both efficacy and cost.

## 5. Conclusions

The ISA78VG-based adjuvant formulations, including ISA78VG&Quil-A, ISA78VG&CpG&MPLA, and ISA78VG&CpG&Quil-A, induced strong antibody responses. Additionally, these formulations provided a broad cross-protection against the H5N8-20135 AIV strain. The ISA78VG&Quil-A combination, in particular, triggered the early antibody production and robust immune responses in SPF chickens, highlighting its potential as a highly effective adjuvant to boost the immunogenicity of the rHA protein subunit vaccine against the highly pathogenic AIV H5N6-20053. Moreover, the relatively low production cost of the ISA78VG&Quil-A formulation makes it an appealing option for large-scale poultry vaccine production, offering a cost-effective solution to control avian influenza outbreaks. In conclusion, these findings underscore the significant promise of ISA78VG-based adjuvant formulations in enhancing both the efficacy and accessibility of vaccines against the avian influenza.

## Figures and Tables

**Table 1:** Antigen and adjuvant immunization regimens.

Group	Antigen (μg)	Adjuvant (μg)				Emulsion (μL)
	rHA	CpG	MPLA	Imiquimod	Quil-A	ISA78VG
1	50					150
2	50	25				150
3	50		20			150
4	50			100		150
5	50				50	150
6	50	25	20			150
7	50	25			50	150
8	-	-	-	-	-	-

1: ISA78VG group. 2: ISA78VG&CpG group. 3: ISA78VG&MPLA group. 4: ISA78VG&Imiquimod group. 5: ISA78VG&Quil-A group. 6: ISA78VG&CpG&MPLA group. 7: ISA78VG&CpG&Quil-A group. 8: PBS group.

**Table 2:** Cytokine primers for qRT-PCR.

Gene	Primer Sequence (5′-3′)	Product Size
IFN-γ	F: ACCTTCCTGATGGCGTGAAG	102 bp
	R: TGAAGAGTTCATTCGCGGCT	
IL-4	F: ATGACATCCAGGGAGAGGTTT	235 bp
	R: ATTGGAGTAGTGTTGCCTGCT	
β-actin	F: TGGGTATGGAGTCCTGTGGT	136 bp
	R: CTGTCAGCAATGCCAGGGTA	

**Table 3:** Viral shedding in chickens challenged by H5N6-20053 virus.

Group	Oropharyngeal Swab (Virus Shedding Number/Total Number)			Cloacal Swab (Virus Shedding Number/Total Number)			Total Virus Shedding Number/Total Number
	3 dpc	5 dpc	7 dpc	3 dpc	5 dpc	7 dpc	
ISA78VG	2/7	0/7	0/7	0/7	0/7	0/7	2/7
ISA78VG&CpG	0/7	0/7	0/7	0/7	0/7	0/7	0/7
ISA78VG&MPLA	0/7	0/7	0/7	0/7	0/7	0/7	0/7
ISA78VG&Imiquimod	2/7	1/7	1/7	0/7	0/7	0/7	3 ^a^/7
ISA78VG&Quil-A	0/7	0/7	0/7	0/7	0/7	0/7	0/7
ISA78VG&CpG&MPLA	0/7	0/7	0/7	0/7	0/7	0/7	0/7
ISA78VG&CpG&Quil-A	0/7	0/7	0/7	0/7	0/7	0/7	0/7
PBS	-	-	-	-	-	-	-

^a^, on day 3, two chickens shed the virus, while on day 5 and day 7, another chicken was detected to be shedding the virus. Therefore, a total of three distinct chickens were observed to have shed the virus. dpc, days post-challenge.

**Table 4:** Positive conversion rate of serum from vaccinated chickens.

Group	Positive Conversion Rate (%)				
	8 d	10 d	12 d	14 d	16 d
ISA78VG&MPLA	0	0	0	60	80
ISA78VG&Quil-A	0	20	80	100	100
PBS	0	0	0	0	0

d, post-vaccination.

**Table 5:** Protective efficacy of the combination adjuvant vaccines against highly pathogenic H5N6-20053 AI viruses in 3-week-old chickens.

Group	HI Titer with Different Antigens (log2)		Virus Shedding from Chickens at Five Days Post-Challenge		Mortality
	rHA	H5N6-20053	Oropharyngeal	Cloacal	
ISA78VG&MPLA	7.8	6.8	0/5	0/5	0/5
ISA78VG&Quil-A	9.8	8.0	0/5	0/5	0/5
PBS	2	0	-	-	-

## Data Availability

The data presented in this study are available upon request from the corresponding author.

## Supplementary Materials

The following supporting information can be downloaded at: https://www.mdpi.com/article/10.3390/vaccines12121448/s1, Figure S1: Hemagglutination activity assay of the H5N6-20053 rHA protein.
