# Supplementary material for: Combination Adjuvants Enhance Recombinant H5 Hemagglutinin Vaccine Protection Against High-Dose Viral Challenge in Chickens
Source: Vaccines (Basel). 2024 Dec 23;12(12):1448. doi: 10.3390/vaccines12121448 (PMC11680309; doi:10.3390/vaccines12121448)
Supplement: Supplementary file 1 [file vaccines-12-01448-s001.zip › vaccines-3350047-supplementary.pdf]

The result showed that the hemagglutination titers of the H5N6-20053 rHA protein were both 12log2 (Figure S1).

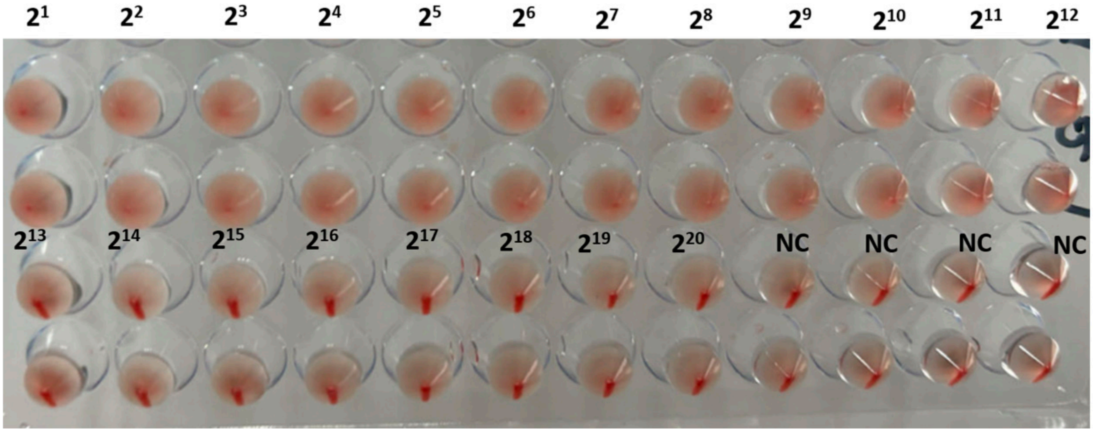

Figure S1. Hemagglutination activity assay of the H5N6-20053 rHA protein.
